# Acute Encephalitis Hospitalizations, California, 1990–1999: Unrecognized Arboviral Encephalitis?

**DOI:** 10.3201/eid1008.030698

**Published:** 2004-08

**Authors:** Rosalie T. Trevejo

**Affiliations:** * Western University, Pomona, California, USA

**Keywords:** epidemiology, encephalitis, arbovirus infections, surveillance, California, research

## Abstract

Historically, Western equine encephalomyelitis and St. Louis encephalitis caused substantial human and equine illness and death in California. This study describes the epidemiology of encephalitis with data from 13,807 patients hospitalized in California with acute encephalitis from 1990 through 1999. The incidence of encephalitis hospitalizations decreased over this period. The greatest proportion of case-patients was hospitalized in the winter. Encephalitis of unspecified origin was the most common diagnosis, and arboviral encephalitis was the least common. Some California counties had concurrent increases in encephalitis rates and in detected arboviral activity in sentinel chickens and mosquito vectors. However, a diagnosis of arboviral encephalitis was made for few hospitalized patients in these counties during these periods. Although some arboviral encephalitis cases may have been undiagnosed, such cases probably did not occur frequently. Active hospital-based surveillance during periods of heightened sylvatic arboviral activity could increase detection of arboviral encephalitis.

## Abstract 2

Epidemiologic features of hospitalized patients provide little evidence of unrecognized arboviral encephalitis.

Encephalitis, or inflammation of the brain, is a serious clinical syndrome with many potential infectious, postinfectious, and postimmunization causes (1,2). Recognized causes of infectious encephalitis in humans include, but are not limited to, herpes simplex viruses, arboviruses, lymphocytic choriomeningitis, mumps, cytomegalovirus, Epstein-Barr virus, human herpesvirus 6, and enteroviruses. The epidemiology of encephalitis in the United States is characterized by the predominance of cases with unknown origin (3–6).

Historically, Western equine encephalomyelitis (WEE) and St. Louis encephalitis (SLE) viruses (WEEV and SLEV, respectively) were important causes of encephalitis in California residents, particularly in the Central Valley and southern California (7). Since the 1960s, the incidence of WEE and SLE has decreased dramatically, although sporadic cases are still reported (7). Most recently, two small SLE epidemics were reported in Los Angeles and the Central Valley in 1984 and 1989, respectively (8,9). Reports of arboviral encephalitis cases are uncommon in southern California, despite evidence of endemic WEEV and SLEV activity in birds and mosquito vectors in that area (10). Sylvatic West Nile virus (WNV) activity was recently detected for the first time in California, which may contribute to changes in the epidemiology of central nervous system disease (11). The 1999 appearance of WNV in New York and adjacent states produced illness and death among humans, horses, and several avian species (12).

Healthcare providers and diagnostic laboratories in California are required to report human encephalitis cases to the California Department of Health Services (CDHS) under Title 17 of the California Code of Regulations. The code stipulates that the reporter identify the cause as viral, bacterial, fungal, or parasitic. Because this surveillance system is passive, human encephalitis cases may be underreported, even when the cause of encephalitis is identified (5). In this study, hospital discharge data were used to estimate the incidence of acute encephalitis and to provide a basis for comparison with the number of reported cases of encephalitis. In addition, the encephalitis hospitalization rates of districts with differing levels of sylvatic arboviral activity were compared. These data may provide a useful baseline to evaluate trends in encephalitis hospitalizations in California, including unusual occurrences of arboviral encephalitis. Such baseline data may prove useful given the recent detection of WNV in California and the potential for introduction of other arboviral agents.

## Materials and Methods

### Data Sources

Hospital discharge data (public use version A) include information on approximately 3.5 million yearly discharges from all California hospitals that serve the civilian population; federal facilities or state hospitals for patients with mental disorders or developmental disabilities are excluded (13). The data do not contain patient names or other personal identifiers. Patients discharged from acute-care hospitals in California from 1990 through 1999 were the source population for the present study. Patients with acute infectious or unspecified encephalitis as the principal diagnosis or one of the 24 additional diagnoses were selected by using the International Classification of Diseases, 9th Revision, Clinical Modification (ICD-9-CM codes listed in Table 1 [14]). Only data on the first hospitalization of patients with more than one encephalitis-related hospitalization were included in the analysis. When available, the record linkage number, based on an encrypted Social Security number, was used to identify patients with multiple hospitalizations. Patients with no record linkage number were assumed to have been hospitalized only once.

**Table 1:** Diagnoses for acute infectious or unspecified encephalitis among hospitalized patients in California, 1990–1999^a^

Diagnosis (ICD-9-CM^b^)	No. of encephalitis diagnoses (%)^c^
Encephalitis of unspecified cause	
Unspecified cause of encephalitis (323.9)	4,841 (34.7)
Unspecified non-arthropod-borne viral diseases of CNS (049.9)	2,932 (21.0)
Viral encephalitis with specified cause, not arboviral	
Acute paralytic poliomyelitis specified as bulbar (045.0)	44 (0.3)
Other specified nonarthropodborne viral diseases of central nervous system (049.8)	288 (2.1)
Herpetic meningoencephalitis (054.3)	2,007 (14.4)
Encephalomyelitis due to rubella (056.01)	6 (0.04)
Rabies (071)	13 (0.1)
Mumps encephalitis (072.2)	14 (0.1)
Encephalitis in viral diseases classified elsewhere (323.0)	76 (0.5)
Other causes of encephalitis	
Other encephalitis due to infection classified elsewhere (323.4)	117 (0.8)
Other causes of encephalitis (323.8)	2,196 (15.8)
Postinfectious causes of encephalitis	
Postvaricella encephalitis (052.0)	421 (3.0)
Post measles encephalitis (055.0)	41 (0.3)
Postinfectious encephalitis (323.6)	595 (4.3)
Bacterial/rickettsial causes of encephalitis	
Tuberculous encephalitis or myelitis (013.6)	30 (0.2)
Meningococcal encephalitis (036.1)	128 (0.9)
Syphilitic encephalitis (094.81)	11 (0.1)
Encephalitis in rickettsial diseases classified elsewhere (323.1)	0 (0)
Parasitic/protozoal causes of encephalitis	
Meningoencephalitis attributable to toxoplasmosis (130.0)	82 (0.6)
Meningoencephalitis attributable to *Naegleria* (136.2)	10 (0.1)
Encephalitis in protozoal diseases classified elsewhere (323.2)	4 (0.03)
Arthropodborne viral encephalitis	
Mosquitoborne viral encephalitis (062.0–062.9)	63 (0.5)
Tickborne viral encephalitis (063.0–063.9)	6 (0.04)
Viral encephalitis transmitted by other and unspecified arthropods (064)	14 (0.1)
Total no. of encephalitis diagnoses^d^	13,939
^a^Patients with a concurrent diagnosis of AIDS are excluded. ^b^International Classification of Diseases, 9th Revision, Clinical Modification. ^c^Source: Office of Statewide Health Planning and Development, Patient Discharge Data, Public Version A. ^d^Total number of encephalitis diagnoses is greater than the number of encephalitis patients (n = 13,807) because some patients had two or more encephalitis diagnoses.	

The annual number of reported encephalitis cases from 1990 through 1999 was obtained from CDHS, Division of Communicable Disease Control. Arboviral surveillance data were obtained from published reports (15–17).

## Data Analysis

The average age- and sex-specific incidence rates of encephalitis (cases per 10^5^ person-years) were calculated by using the California population projections for 1995 (18). These estimates were adjusted to account for the proportion of cases with missing demographic information by assuming that the proportion with missing data is the same within each subgroup. A 95% confidence interval was calculated for each group-specific rate. Such rates were compared by using a chi-square test for proportions. Annual incidence rates were calculated by using annual population estimates for California (19). Linear trends in proportions were evaluated by using a chi-square test for trend (20). Significance probabilities <0.05 (p values) were considered a strong indication of systematic influence (i.e., not chance variation).

Encephalitis hospitalization rates were evaluated separately for Sacramento and Yolo, Sutter and Yuba, and Riverside and Imperial Counties. These counties had a sufficient population size to allow calculation of meaningful rates and had reported sylvatic arboviral activity during the study period. The number of encephalitis cases was insufficient to calculate county-specific rates for each category of encephalitis and therefore the rates are for all encephalitis hospitalizations. Data were combined for Sacramento-Yolo and Sutter-Yuba to reflect the collection of arboviral surveillance data by a single bi-county agency for each of these districts, and for Riverside-Imperial to reflect their proximity and similarity in sylvatic arboviral activity. Temporal trends in encephalitis rates from 1991 through 1999 were examined in conjunction with arboviral surveillance data on sentinel chicken seroconversions to WEEV and SLEV and on virus-positive mosquito pools. The county-specific encephalitis rates for years with increased sylvatic arboviral activity were compared with the average rate for the remaining years by using a two-sample test for equality of proportions. The county of residence for patients hospitalized in 1990 was not available, precluding the inclusion of data from that year in the county-level analyses. Analyses were conducted with EpiInfo 6 (version 6.04d; Centers for Disease Control and Prevention, Atlanta, GA), SAS (version 8; SAS Institute, Cary, NC), and S-Plus 2000 (Professional Release 3; MathSoft, Inc., Seattle, WA).

## Results

**Figure 1:**
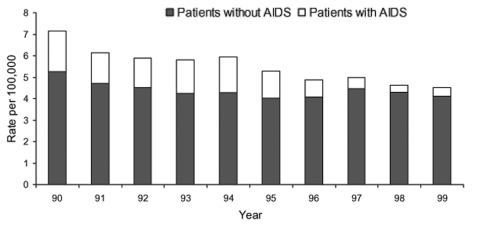
Cumulative incidence of encephalitis hospitalizations in California, 1990–1999 (n = 17,318).

From 1990 through 1999, a total of 17,318 patients were hospitalized with acute encephalitis; 3,511 (20.3%) had a concurrent diagnosis of AIDS. The proportion of encephalitis patients per year with AIDS decreased from 27% in 1990 to 9.5% in 1999 (chi-square test for trend = 475.9, p < 0.001). Annual rates for patients with and without AIDS are shown in Figure 1. All subsequent analyses were limited to the 13,807 patients without a concurrent diagnosis of AIDS because of the distinct epidemiologic characteristics of these two populations.

Unspecified encephalitis made up most of the encephalitis diagnoses (55.7%), followed by specified viral encephalitis (not arboviral) (17.6%) and "other" causes of encephalitis (16.6%) (Table 1). Arthropodborne viral disease constituted <1% of the encephalitis diagnoses, with a total of 83 diagnoses among 82 patients. Some patients had more than one ICD-9-CM code for encephalitis. Thus, the total number of diagnoses was greater than the number of patients (Table 1).

The encephalitis rate was highest in infants (<1 year old), followed by persons >65 years of age (Table 2). The lowest rate was in persons 20–44 years of age. The chi-square test for proportions indicated that the difference in the rates between each age group was significant. Female patients had a rate that was significantly higher than that of male patients.

**Table 2:** Characteristics of patients hospitalized with acute infectious or unspecified encephalitis, California, 1990–1999^a^

	No. of patients^b^	1995 population^c^	Rate (95% CI)^d^
Sex			
Male	6,684	16,062,552	4.2 (4.1–4.3)
Female	7,123	16,000,360	4.5 (4.3–4.6)
Age group, y			
<1	868	552,649	15.7 (14.7–16.8)
1–4	973	2,356,048	4.1 (3.9–4.4)
5–19	2,350	6,801,354	3.5 (3.3–3.6)
20–44	4,157	12,964,498	3.2 (3.1–3.3)
45–64	2,707	5,958,743	4.5 (4.4–4.7
65+	2,752	3,429,620	8.0 (7.7–8.3)
Overall	13,807	32,062,912	4.3 (4.2–4.4)

^a^Patients with a concurrent diagnosis of AIDS are excluded. ^b^Source: Office of Statewide Health Planning and Development, Patient Discharge Data, Public Version A. ^c^Population projections, State of California, Department of Finance. ^d^Cases per 10^5^ person-years = (frequency/[1995 population x 10]) x 100,000; CI = confidence interval.

**Figure 2:**
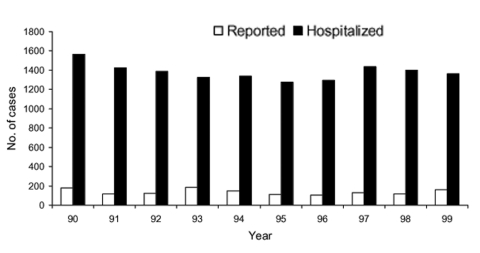
Comparison of hospitalized versus reported encephalitis in California, 1990–1999. Hospitalized patients with a concurrent diagnosis of AIDS were excluded.

A comparison of the annual number of patients hospitalized with encephalitis with the number of encephalitis cases reported to CDHS is shown in Figure 2. On average, the number of patients hospitalized with acute infectious or unspecified encephalitis was 10-fold higher than the number of encephalitis cases reported to CDHS.

The epidemiology of encephalitis in specific counties with sylvatic arboviral activity over the study period was further examined to evaluate the potential role of undiagnosed arboviral encephalitis. Most patients hospitalized with encephalitis in Sacramento-Yolo, Sutter-Yuba, and Imperial-Riverside Counties were diagnosed with unspecified encephalitis (Table 3). Only 10 patients were diagnosed with arthropodborne viral encephalitis, 2 from Sacramento-Yolo, 1 from Sutter-Yuba, and 7 from Riverside-Imperial. The number of admissions per quarter was distributed fairly evenly for each bi-county area when the data from 1991 through 1999 were combined (Table 3). In no case did the proportion of hospitalizations for any given quarter differ significantly from the null value of 25% (one-sample test for proportions: p > 0.05).

**Table 3:** Diagnoses and month of admission for patients hospitalized with acute infectious or unspecified encephalitis in selected California counties, 1991–1999

	Sacramento-Yolo	Sutter-Yuba	Imperial- Riverside
1995 Population estimates	1,271,500	135,400	1,500,300
Diagnosis (ICD-9-CM^a^)	No. of encephalitis diagnoses (%)^b^		
Encephalitis of unspecified cause	305 (60.2)	45 (70.3)	338 (58.2)
Viral encephalitis with specified cause, not arboviral	74 (14.6)	11 (17.2)	112 (19.4)
Other causes of encephalitis	76 (15.0)	5 (7.8)	64 (11.1)
Postinfectious causes of encephalitis	38 (7.5)	1 (1.6)	45 (7.8)
Bacterial/rickettsial causes of encephalitis	10 (2.0)	0 (0)	7 (1.2)
Parasitic/protozoal causes of encephalitis	2 (0.4)	1 (1.6)	5 (0.09)
Arthropodborne viral encephalitis^c^	2 (0.4)	1 (1.6)	7 (1.2)
Total no. of encephalitis diagnoses^d^	507	64	578
Month of hospital admission	No. of admissions (%)		
January–March	112 (22.1)	17 (26.6)	160 (28.0)
April–June	126 (24.9)	12 (18.8)	127 (22.2)
July–September	139 (27.5)	15 (23.4)	140 (24.5)
October–December	129 (25.5)	20 (31.3)	145 (25.3)
Overall	506 (100)	64 (100)	572 (100)

^a^International Classification of Diseases, 9th Revision, Clinical Modification. ^b^Source: Office of Statewide Health Planning and Development, Patient Discharge Data, Public Version A. ^c^Arboviral encephalitis was diagnosed in Sacramento in 1992 (n = 1) and 1997 (n = 1), in Yuba in 1999 (n = 1), in Imperial in 1997 (n = 1), and in Riverside in 1991 (n = 3), 1994 (n = 2), and 1997 (n = 1). ^d^Total number of encephalitis diagnoses in Sacramento-Yolo and Imperial-Riverside is greater than the number of encephalitis patients because some patients had two or more encephalitis diagnoses.

**Figure 3:**
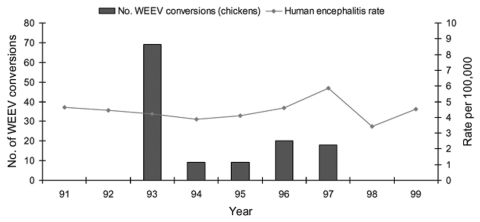
Annual rate of encephalitis hospitalizations and annual number of sentinel chicken seroconversions to Western equine encephalomyelitis virus (WEEV) infection, Sacramento and Yolo Counties, California, 1991–1999. Hospitalized patients with a concurrent diagnosis of AIDS were excluded.

**Figure 4:**
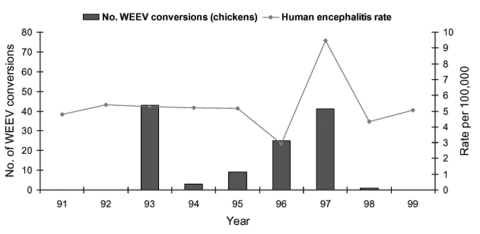
Annual rate of encephalitis hospitalizations and annual number of sentinel chicken seroconversions to Western equine encephalomyelitis virus (WEEV), Sutter and Yuba Counties, California, 1991–1999. Hospitalized patients with a concurrent diagnosis of AIDS were excluded.

**Figure 5:**
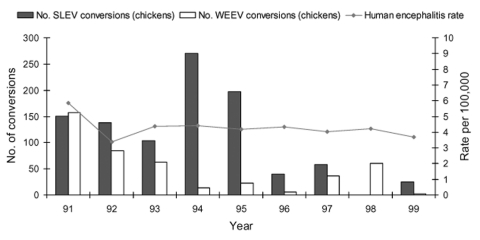
Annual rate of encephalitis hospitalizations and annual number of sentinel chicken seroconversions, Imperial and Riverside Counties, California, 1991–1999. Hospitalized patients with a concurrent diagnosis of AIDS were excluded. SLEV, St. Louis encephalitis virus; WEEV, Western equine encephalitis virus.

Annual encephalitis rates are shown for Sacramento-Yolo (Figure 3), Sutter-Yuba (Figure 4), and Imperial-Riverside (Figure 5) Counties from 1991 through 1999. The encephalitis rates in these areas increased during some years when increased arboviral activity was detected in sentinel chickens flocks and mosquito pools (Table 4). For instance, the encephalitis rate in Sutter-Yuba increased in 1997 (two-sample test for proportions: p = 0.041), when 41 sentinel chickens seroconverted to WEEV. A smaller increase in the encephalitis rate was observed in Sacramento-Yolo in 1996 and 1997 (two-sample test for proportions: p = 0.028), when 20 and 18 sentinel chickens seroconverted to WEEV, respectively. In contrast, the encephalitis rates did not increase in Sacramento-Yolo and Sutter-Yuba in 1993, when increased WEEV activity was detected in sentinel chickens and mosquito pools. In Riverside-Imperial, a small increase in the encephalitis rate in 1991 (two-sample test for proportions, p = 0.004) corresponded with an increase in detected WEEV and SLEV activity in sentinel chickens and mosquito pools. The proportion of encephalitis hospital admissions was higher than expected (>25%) during the summer (July–September) in Sutter-Yuba in 1997 (9/13 [69.2%]) and in Imperial-Riverside in 1991 (48/161 [29.8%]) (one-sample test for proportions: p = 0.008 and p = 0.187, respectively). No such increase occurred in Sacramento-Yolo in 1996 and 1997 (34/135 [25.2%]).

**Table 4:** SLEV- and WEEV-positive *Culex tarsalis* pools in selected California counties, 1991–1999^a,b^

Year	No. of virus-positive mosquito pools (no. pools tested)^c^			
	Sacramento-Yolo	Sutter-Yuba	Imperial-Riverside	
	WEEV	WEEV	WEEV	SLEV
1991	0	0	73	44
1992	0	0	20	10
1993	81	31	13	15
1994	4	0	1	7
1995	2	1	0	0
1996	9	2	0	1
1997	10	14	0	0
1998	0 (187)	0 (172)	11 (968)	1 (968)
1999	0 (548)	0 (232)	0 (918)	0 (918)

^a^SLEV, St. Louis encephalitis virus; WEEV, Western equine encephalitis virus. ^b^Sources: Hui et al., 1999 (1991–1997); CDHS, Vector-Borne Disease Section, Annual Reports (1998 & 1999). ^c^The number of mosquito pools tested (denominator) was only available for 1998 and 1999.

## Discussion

The encephalitis rate showed an overall decrease during the study period. The high proportion of patients with unspecified encephalitis in this study (Table 1) is consistent with findings from other studies and raises questions about potential causes of these encephalitis cases (3,5). Arboviral encephalitis was diagnosed in <1% of patients hospitalized with acute encephalitis from 1990 through 1999. This finding indicates that this type of encephalitis is either exceedingly rare in California or underdiagnosed. In the absence of public health alerts during periods of epizootic arboviral activity, clinicians may be disinclined to pursue laboratory testing for arboviral agents because of a low index of suspicion. Furthermore, outside of academic interest, clinicians may not have much incentive to request laboratory testing for specific agents for patients with viral encephalitis if a specific diagnosis will not change the course of treatment.

ICD-9-CM codes used in the hospital discharge database provide a standardized means of comparing data between hospitals. A previous study used ICD-9-CM codes in the National Hospital Discharge Survey to describe the epidemiology of encephalitis (4). In both studies, the age-specific encephalitis rates were highest among infants (<1 year) and the elderly (>65 years) (Table 2). This finding may be due in part to infection with herpes simplex, which is a common cause of nonepidemic, acute encephalitis that occurs most frequently in children and the elderly (6). Patients with AIDS, 92.5% of whom were men, were excluded in the present study, which likely resulted in a higher proportion of females compared to the national study.

An advantage of using hospital discharge data to study the epidemiology of encephalitis is that most patients with encephalitis are likely to be hospitalized because of the severity of the illness. Accordingly, these findings are more readily generalized to the population of California, unlike the passive surveillance data, which are limited by underreporting (21). In the present study, the annual number of hospitalized encephalitis patients was approximately 10-fold greater than the annual number of reported cases (Figure 2). The actual degree of underreporting may be less, as not all of the hospitalizations for encephalitis may have been due to reportable causes. However, evidence exists that arboviral encephalitis was underreported in California from 1990 through 1999, with 82 patients hospitalized with arthropodborne viral encephalitis but only 7 arboviral encephalitis cases reported. Encephalitis cases with an unspecified cause may also be disproportionately unreported, since encephalitis cases reported under the current passive surveillance system request that the reporter specify the cause as viral, bacterial, fungal, or parasitic.

A disadvantage of relying on the public use hospital discharge dataset to describe the epidemiology of encephalitis is the lack of patient identifiers. This fact may have resulted in multiple hospitalizations for individual patients being included, as evidenced by the number of hospitalizations for poliomyelitis and rabies (Table 1). Another disadvantage is the lack of information on laboratory test results used to make diagnoses. While the high proportion of unspecified encephalitis cases in the present study possibly resulted from underuse of appropriate diagnostic tests, other studies do not support this hypothesis. For instance, from 1956 through 1958, a total of 1,595 encephalitis patients were identified in Kern County through active hospital-based surveillance and evaluated by using a standard battery of tests (5). No cause was identified for 569 (36%) patients, and WEE and SLE accounted for <5% of cases per year. When advanced diagnostic methods were used, the cause of encephalitis was identified for only 126 (38%) of 334 patients referred to the California Encephalitis Program from June 1998 through December 2000; no patients with arboviral encephalitis were identified (3). These findings raise the possibility that current diagnostic tests may simply be inadequate for identifying all possible causes of encephalitis.

Given the limited number of encephalitis hospitalizations in any given county, all encephalitis diagnoses were combined to provide a meaningful examination of county-specific trends. Combining these diagnoses may have obscured trends in specific disease agents, although most of the patients in Sacramento-Yolo, Sutter-Yuba, and Riverside-Imperial had a diagnosis of unspecified encephalitis (Table 3). In Sacramento-Yolo and Sutter-Yuba, concurrent increases in sylvatic WEEV transmission and in the rates of encephalitis hospitalizations in 1997 occurred (Table 4; Figures 3 and 4), increasing the likelihood that a proportion of the unspecified encephalitis cases may have been due to arboviral encephalitis. A similar pattern was observed in Imperial and Riverside Counties in 1991, when levels of sylvatic WEEV and SLEV transmission were particularly high (Table 4 and Figure 5). The proportion of hospital admissions for encephalitis in Sutter-Yuba in 1997 and Imperial-Riverside in 1991 increased during the summer months, when arboviral and enteroviral transmission most commonly occur (1). In contrast, statewide hospital admissions for encephalitis were significantly higher during the winter months, which indicates that arboviral encephalitis is typically an unimportant contributor to encephalitis hospitalizations. A study of encephalitis patients in California from 1956 through 1958 also found an unexplained increase in the proportion of cases with undetermined origin during the winter (5). One possible cause, lymphocytic choriomeningitis, occurs most commonly in the winter, although it is thought to be rare (1).

The arboviral surveillance data used in the present study lacked denominator data on mosquito pools and sentinel chicken specimens tested for most years. In addition, mosquito pool and sentinel chicken surveillance is not uniform across mosquito control districts. In spite of these limitations, notable increases in arboviral activity in mosquito pools (Table 4) and sentinel chickens (Figures 3–5) were observed in some years. Many potential reasons exist for the lack of consistent correlation between sylvatic arboviral activity and encephalitis rates. One possibility is that mosquito population indices, sylvatic arboviral transmission levels, or both, were not always sufficient to increase the risk for human infection. For instance, *Culex tarsalis* population indices are correlated with sentinel chickens seroconversion rates for WEEV and SLEV (22). A retrospective study of a 1989 SLE epizootic in the Central Valley, with high *Cx. tarsalis* abundance, 70 virus-positive mosquito pools, and seroconversion of 71% of sentinel chickens, identified 28 (43%) of 65 aseptic meningitis and encephalitis patients as SLE patients (9,23). In the present study, the encephalitis rate in Imperial-Riverside increased slightly in 1991, when WEEV and SLEV activity was detected in many mosquito pools and sentinel chickens, but remained relatively unchanged in 1994 and 1995, when many sentinel chickens seroconverted but few mosquito pools were virus-positive. Another possible contributor to the study findings is variation in the virulence of circulating arboviruses over the study period. Three phenotypes of WEEV, which differed in their virulence properties in adult mice, were isolated from mosquito pools collected in California from 1991 through 1995 (24). Lastly, sylvatic arboviral activity and encephalitis rates may not be correlated. For instance, no encephalitis cases were detected during an intense WNV epizootic in Connecticut (25). In fact, an aseptic meningitis epidemic attributable to enteroviruses was detected during an avian epizootic of WNV, while no WNV meningitis cases were detected (26).

Many factors could explain the observed decrease in the incidence of clinical WEE and SLE cases since the 1960s. Mosquito control and water management programs have been effective at reducing mosquito vector populations (27). Changes in human behavior may also have coincided with the decrease in the incidence of arboviral illness (28). With the advent of television and air-conditioning, people are more likely to remain indoors during twilight hours, when peak feeding by vector species takes place. Earlier research showed rural residents to be at higher risk for arboviral illness than urban residents (29). With changes in land use over the past century, a greater proportion of the human population now resides in urban and suburban settings. A 1995 California study of outpatients attending county health department clinics found a significantly higher seroprevalence of WEEV among residents of rural Imperial and Sutter Counties than Sacramento County residents; the seroprevalence for SLEV was significantly higher in Imperial than in both Sacramento and Sutter Counties (30). However, although this study was conducted in areas with both sporadic and enzootic WEEV and SLEV transmission, the overall seroprevalence levels for both viruses were low.

The methods used in the present study are useful for evaluating trends in the incidence of emerging or potentially emerging diseases. The epidemiology of arboviral encephalitis will likely change with the establishment of WNV in California, making active hospital-based surveillance for arboviral disease an important supplement to traditional passive reporting. These findings suggest that unrecognized arboviral encephalitis has not constituted a large proportion of the unspecified encephalitis patients who were hospitalized from 1991 through 1999. However, the study results do indicate the potential utility of intensified surveillance efforts during periods of increased sylvatic arboviral activity. During such periods, implementing active hospital-based surveillance for encephalitis, acute flaccid paralysis, and aseptic meningitis, with collection and testing of diagnostic specimens, may result in detecting cases of arboviral disease that would otherwise go undiagnosed and unreported.
